# The future of endangered crayfish in light of protected areas and habitat fragmentation

**DOI:** 10.1038/s41598-020-71915-w

**Published:** 2020-09-10

**Authors:** Lucian Pârvulescu, Elena-Iulia Iorgu, Claudia Zaharia, Mihaela C. Ion, Alina Satmari, Ana-Maria Krapal, Oana-Paula Popa, Kristian Miok, Iorgu Petrescu, Luis-Ovidiu Popa

**Affiliations:** 1 Department of Biology-Chemistry, Faculty of Chemistry, Biology, Geography, West University of Timisoara, 300115 Timisoara, Romania; 2 “Grigore Antipa” National Museum of Natural History, 011341 Bucharest, Romania; 3 Department of Mathematics, Faculty of Mathematics and Computer Science, West University of Timisoara, 300223 Timisoara, Romania; 4 Institute of Biology Bucharest, Romanian Academy, 060031 Bucharest, Romania; 5 Faculty of Biology, University of Bucharest, 050095 Bucharest, Romania; 6 Department of Geography, Faculty of Chemistry, Biology, Geography, West University of Timisoara, 300223 Timisoara, Romania; 7 Department of Informatics, Faculty of Mathematics and Computer Science, West University of Timisoara, 300223 Timisoara, Romania

**Keywords:** Ecology, Conservation biology, Ecological genetics, Ecological modelling, Ecological networks, Freshwater ecology

## Abstract

The long-term survival of a species requires, among other things, gene flow between populations. Approaches for the evaluation of fragmentation in the frame of freshwater habitats consider only a small amount of the information that combined demography and geography are currently able to provide. This study addresses two species of *Austropotamobius* crayfish in the light of population genetics, spatial ecology and protected areas of the Carpathians. Advancing the classical approaches, we defined ecological distances upon the rasterised river network as a surrogate of habitat resistance to migration, quantifying the deviations from the species´ suitability range for a set of relevant geospatial variables in each cell of the network. Molecular analyses revealed the populations of the two *Austropotamobius* crayfish species are clearly distinct, lacking hybridisation. Comparing pairs of populations, we found, in some cases, a strong disagreement regarding genetic and ecological distances, potentially due to human-mediated translocations or the geophysical phenomena of regressive erosion, which may have led to unexpected colonisation routes. Protected areas were found to offer appropriate local habitat conditions but failed to ensure connectivity. The methodology applied in this study allowed us to quantify the contribution of each geospatial (environmental) variable to the overall effect of fragmentation, and we found that water quality was the most important variable. A multilevel approach proved to reveal a better understanding of drivers behind the distribution patterns, which can lead to more adequate conservation measures.

## Introduction

For species with insular distributions, conservation measures through the establishment of Protected Areas (PAs) are only partially useful in the long run, as these measures usually fail to ensure the connectivity between populations^1,2^. Connectivity for freshwater organisms depends on the physical distance between the populations on the river path as well as the quality of habitats this path traverses^3^. The success of efforts to establish corridors for migration, especially in case of rare and sensitive species, is substantially dependent on the accuracy of ecological assessment methods employed^4,5^. Fragmentation is one of the most challenging issues in conservation biology because its assessment is based on the evaluation of a wide array of interlocking factors^4,6^. Current techniques for molecular analysis can accurately pinpoint the degree of separation between populations, but the tools for measuring the ecological fragmentation within hydrological networks are still underdeveloped.

In the context of increasing global human demand^7,8^ and pressure on freshwater systems^9,10^, the future of biodiversity is marked by the question whether PAs are able to face the next challenges in ecosystems services^11^. Good management of PAs should focus, among others, on minimising the loss of priority species and their habitat^12,13^ and restoring the connectivity of populations^1,14^. Many crayfish species are protected as ecologically sensitive, requiring long-term habitat stability^15,16^. The most sensitive indigenous European crayfish species in terms of ecological and conservation requirements (see e.g. ref.^17^) are the *Austropotamobius* species: the white-clawed crayfish *A. pallipes* sensu lato (Lereboullet, 1858) and the stone crayfish *A. torrentium* (von Paula Schrank, 1803). Recently, a new *Austropotamobius* species, namely the idle crayfish *A. bihariensis*^18,20^, was described by phylogenetic and taxonomic evidence. Both *A. torrentium* and *A. bihariensis* reside in the Romanian Carpathians^19,20^ and prefer habitats with clean waters in the mountainous and submountainous regions^18,21^. The range of *A. torrentium*^19^ comprises Central Europe and the Balkans, and the metapopulation in Romania is representative for this species as it consists of its most common haplogroup^20,22^. *Austropotamobius bihariensis* is an endemic species restricted to the Apuseni Mountains, Romania^18^.

Multilevel approaches are expected to reveal the relationship between species’ biology and their habitats more accurately^23–25^. Large-scale evaluations in spatial ecology require remotely computable variables rather than pointwise field measurements^26,27^. In our assessment of fragmentation, we started from the idea that, in order for two populations to exchange genes, the individuals need to travel through a sector of river with characteristics that may or may not conform to the species’ ecological requirements^2^. Working on a rasterised river network, we introduced ecological distances, a surrogate of habitat resistance to migration widely used in landscape ecology. The ecological distance between a pair of populations is determined by the deviation from the species suitability range in each cell of the network pathway, for a relevant set of spatial variables. Coupled with population genetics data, this allowed us to obtain a better understanding of the distribution of crayfish and the efficiency of the PAs for their conservation.

## Methods

### Field data

**Figure 1:**
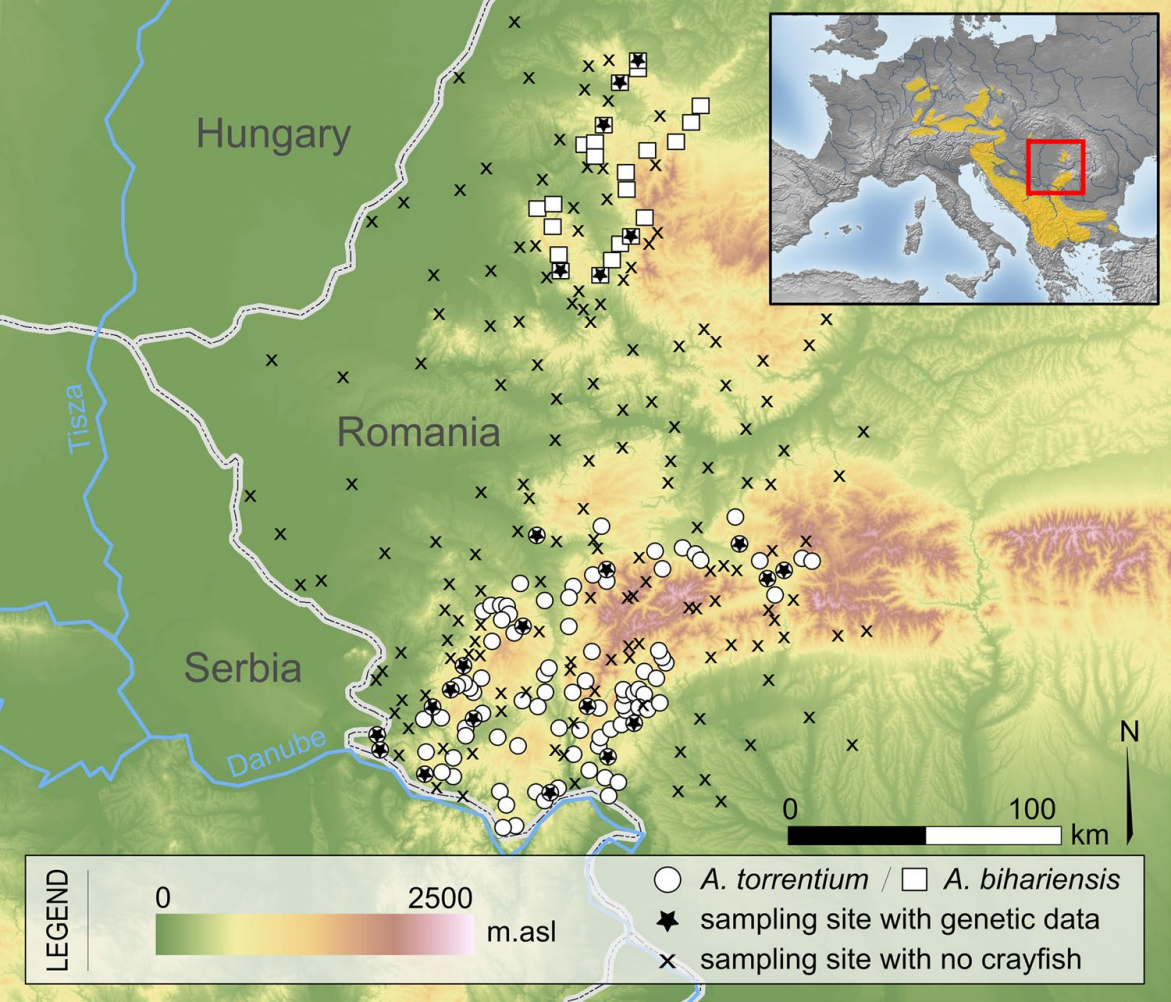
Distribution map of sampling sites in the investigated area. The map was generated by using the ArcGIS version 10.4 software (https://www.esri.com) and designed in Inkscape 0.92.4 (https://inkscape.org).

Survey datasets of presence and absence of *A. torrentium* and *A. bihariensis* in the Romanian Carpathians were obtained from recent published papers^18,21,28^, totalling 274 sites (126 presences and 148 absences, Fig. 1). We considered a population to be all the individuals from one stream, and therefore sampled accordingly. The sampling locations were randomly selected, considering the species’ distributions. In situ, the small tributaries were investigated by hand sampling from the riverbed by thoroughly checking galleries in the riverbanks and spaces between or beneath rocks and roots, comprising approximately 200 m of river stretch, while large rivers where inspected using baited traps (Pirate type, with double entrance), left over night. Local residents were also asked about the presence of crayfish, but this information was considered only if it was confirmed in the field. All the sites used in this study were visited at least twice over the past years, confirming the presence or absence of targeted crayfish. Out of the 274 sites, 50.7% were situated in PAs. Additionally, we sampled 411 individuals from 23 sites (at least 10 individuals from each site) for further genetic analyses (see Fig. 1).

Sample collection for the present study did not significantly affect the wild animals (according to ref.^29^). After one segment from the last pair of walking legs from each individual was detached and stored in 96% ethanol, the animals were released back into their habitat, in compliance with national and international ethical guidelines. This study was approved by the Romanian Academy, Commission for Monuments of Nature Protection (decision no. 3131/08.04.2009), and additionally, licences were granted from the legal administrators of Natura 2000 sites in the investigated area.

### The raster of spatial variables

Four GIS-derived environmental variables (see below) were selected based on existing literature as relevant for *A. torrentium* ecology (e.g. refs.^30–32^). The SRTM V3 void filled ^33^ digital elevation model (DEM) at a 100-m resolution was processed and used for the extraction of the river network of the analysed area in ArcGIS version 10.4 (ArcMap; Environmental Systems Resource Institute, Redlands, California). Each cell (100 × 100 m) of the river network was populated with the values of the four selected geospatial variables as follows.

We used *RIVERenhancer* to remotely estimate water quality by using the variable RWQ as defined by ref.^34^, based on land cover data extracted from remote sensing images. Considering that each cell in the river raster collects environmental influences from its upstream drainage basin, RWQ is defined using the Corine Land Cover (version 18.5.1; https://land.copernicus.eu) categories found in the catchment area of each cell in the river network grouped by anthropogenic influence potential^35^ and weighted by their respective areas in the catchment^34^. Flash-flood potential (FFP) was used to quantify the riverbed stability^32^; it estimates the potential for flood-related disturbance to streams by taking into account the potential drainage velocity both upstream and at the site. This variable was calculated as a product of two land-surface variables, namely slope gradient and catchment slope, derived from the DEM. The multiannual mean temperature (MMT) was extracted from WorldClim global climate layers (www.worldclim.org) raster datasets of 1-km spatial resolution, resampled at 100 m. The altitude (ALT) was extracted directly from the DEM.

### Statistics for the assessment of ecological fragmentation

As seen in Table 1, *A. bihariensis* appears to occupy a slightly narrower ecological niche than *A. torrentium*; its occupied range based on field observations was fully included in that of *A. torrentium* for all the considered variables, but no statistically significant differences were found between the species regarding the distribution of any of the environmental variables (Mann–Whitney, p > 0.1 in all cases). For this reason, and considering the fact that until very recently^18^, the two *Austropotamobius* species were regarded as being one and the same, their records were analysed in a single dataset. The presence/absence data were used to establish the most suitable range of each variable for the species distribution. The values of these characteristics in each point of the raster river network situated outside the species suitability range imposed an “ecological cost”, i.e. a stress that the travelling individuals would have to face (see e.g. ref.^36^). An “ecological distance” between pairs of populations was defined by summing the ecological costs along the river path connecting them.

**Table 1:** Range occupied by *A. torrentium* and *A. bihariensis* (field observations) vs. suitability range predicted by the random forest model for the habitat variables considered (FFP—flash flood potential; RWQ—remote water quality; ALT—altitude; MMT—multiannual mean temperature; % overlap was computed between maximal observed and predicted ranges; *AuT*—*A. torrentium*; *AuB*—*A. bihariensis*).

Variable	FFP	RWQ	ALT	MMT
Sampled range	0–11.447	0–3	3–1,420	3.2–11.8
Observed range *AuT*	0.012–6.014	0–1.04	105–863	7.1–11
Observed range *AuB*	0.14–3.55	0–0.77	277–696	7.9–9.7
Predicted range	0–6.1	0–1	137–950	6.97–10.82
% overlap	98.4	96.1	85.9	92.3

Specifically, a Random Forest classification model was fit to the species presence/absence data, considering RWQ, FFP, ALT and MMT as predictors. We used the *randomForest* package^37^, version 4.6-12, of the R software^38^. In total, 5,000 trees were generated, with two predictors randomly chosen for each split. The cutoff probability for discrimination between classes was determined so that predicted species prevalence would equal observed prevalence^39^ and set at 0.35. Variable importance was assessed by the amount that the Gini index decreased by splits over the respective predictor, averaged over all the trees (Fig. S1). Response curves for habitat variables were obtained as partial dependence plots, illustrating the effect of the selected variable on presence probability after integrating out the other predictors. A species suitability range was then computed for each variable from the response curve, as the range where the predicted probability of presence exceeded the class discrimination threshold. This suitability range was validated against the species-occupied range (as observed in the field), and the two were found to be in high agreement (see Table 1).

Next, the full range of each variable across the analysed river network was linearly mapped to the interval [0,1]. The transformation was performed in order to assign similar importance to each variable in further cost computation. A cost per variable was computed in each cell of the river network as the distance between the scaled value of the variable in that cell and the scaled suitability range. The ecological cost for a cell x was then computed as

$$ecologicalCost\left(x\right)={\left({\sum }_{i}Cos{t}_{i}{\left(x\right)}^{2}\right)}^\frac{1}{2},$$

where the summation is made over all predictor variables *i* (i.e. the ecological cost is the Euclidean distance from the four-dimensional point defined by the scaled values of the habitat variables in that cell to the hyper-rectangle given by the scaled suitability ranges). The considered predictors are of comparable importance in the model (see Fig. S1) and were therefore weighed equally in the computation of costs. The cumulative ecological cost on a path connecting a pair of populations defines the “ecological distance” between those populations.

### Spatial data management

**Figure 2:**
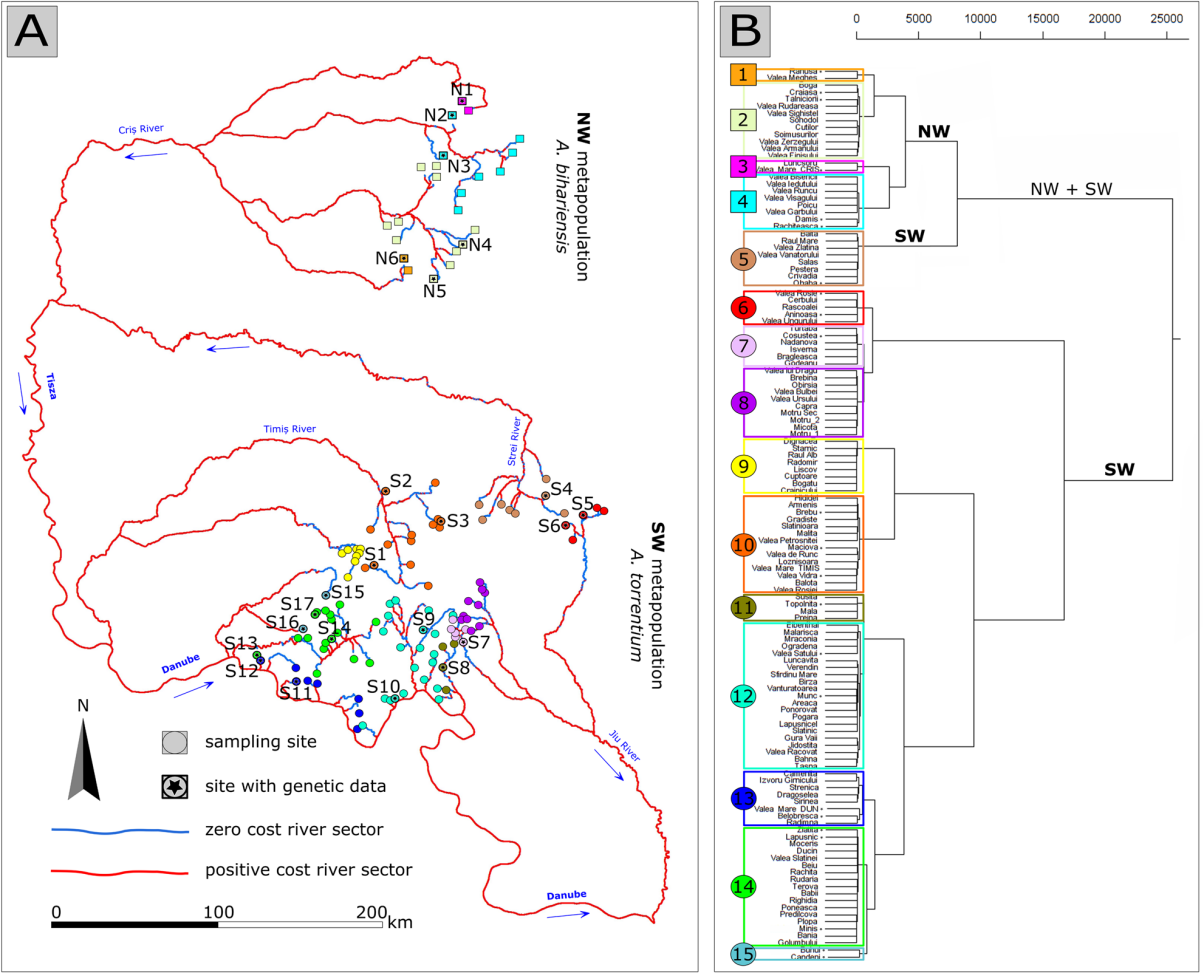
Prediction of suitability of the river network connecting the 126 populations of crayfish **(A)** and clustering based on ecological distances **(B)**. Sites where genetic samples were collected are numbered (N1–6 for northern sites, S1–17 for southern sites).

We used the *network analyst* extension in ArcGIS version 10.4 to connect each of the 126 crayfish populations via the hydrological network to calculate the *origin–destination* matrix for the ecological distance (see Fig. 2A). Hierarchical clustering on the ecological distance matrix was performed using Ward's method to identify population clusters. Visual inspection of the resulting dendrogram showed the presence of 15 well-separated populations clusters.

A new polygon shapefile was created to delineate the population clusters within the river network. Each polygon contained the populations within a cluster, all the cells of the upstream branching network, as well as the downstream cells up to the point where the last tributary with a crayfish population from that cluster flows into the main river.

To assess the influence of protected areas (PAs) on the crayfish distribution pattern, the shapefiles of PAs in the region were superimposed over the river network. Sites of Community Importance in Romania (https://www.mmediu.ro/articol/date-gis/434) and Hungary (https://www.protectedplanet.net/country/HU) were considered for the EU region and PAs in the Republic of Serbia (https://www.protectedplanet.net/country/RS).

Each cell of the river network was finally assigned, based on location, as belonging to one of the 15 ecological clusters or to the river sector connecting those clusters, to PA or outside of PA.

### Population genetics

Microsatellite analysis was performed on samples from 23 populations, of which 17 belonged to *A. torrentium* (South-Western Populations) and 6 to *A. bihariensis* (North-Western Populations). Sample size ranged from 10 to 66 individuals per population. The DNA extraction and PCR conditions followed the protocols described by ref.^40^. We used a set of five polymorphic microsatellites: aas5, aas6, aas3040, ap1^40^ and Tor11, with the following sequence: Tor11F (5′- TGCTGCCCATGATATCCTTT-3′) and Tor11R (5′-TGGGGATGGTCATTTTGTT-3′) (unpublished data).

We ran PCR products on a LI-COR 4300 DNA analyser platform and visualised the results using IRD Dye 700 and 800 and a 50–700-bp size standard (LI-COR Biosciences, Li-COR Inc., Lincoln, NE, USA). The program SAGA v. 3.3 (LI-COR Biosciences, Li-COR Inc., Lincoln, NE, USA) was used to score the microsatellite alleles and to determine the genotypes.

Observed and expected heterozygosity (H_O_ and H_E_), number of alleles and inbreeding coefficient (F_IS_) for each locus and each population were calculated in GenAlEx v. 6.502^41^. Allelic richness (AR) was calculated using FSTAT v. 2.9.3^42^. Deviations from Hardy–Weinberg equilibrium (HWE) and linkage disequilibrium between all pairs of loci were tested in Genepop v. 4.6^43^, and significance was determined using 100 batches and 1,000 iterations per batch.

We used MicroChecker v. 2.2.3^44^ software to check for null alleles, genotyping error rates, allelic dropout or stuttering and to calculate null alleles frequencies. We also estimated null allele frequencies, as well as the corrected FST values using the Expectation Maximization algorithm^45^ and ENA method, implemented in FreeNA^46^.

Genetic differentiation was quantified calculating pairwise F_ST_ values^47^, calculated in GenAlEx v. 6.502; in addition, we compared FST values with and without the correction for null alleles calculated in FreeNA, using a t-test performed in Past v.1.55^48^. We also performed AMOVA to determine how the genetic variation was partitioned within and among populations grouped in the hierarchical schemes according to the clusters inferred from STRUCTURE, using GenAlEx v. 6.502. Isolation by distance was tested using a Mantel test implemented also in GenAlEx v. 6.502. A phylogenetic tree was constructed based on the microsatellite data using Neighbour-Joining algorithm and D_a_ genetic distance^49^, implemented in POPULATIONS v. 1.2.32^50^.

We carried out a Bayesian analysis in STRUCTURE v. 2.3.4^51^ to establish the number of genetic clusters of investigated samples, using all available individuals. For each value of K, from K = 1 to K = 25, we performed 10 independent runs of 750,000 generations, following 250,000 generations of burn-in under the admixture model and with the assumption that allele frequencies among populations are correlated. We identified the optimal number of clusters using both the Evanno method (ΔK; ref.^52^) and the highest probability for K following ref.^51^ on the CLUMPAK online platform (https://clumpak.tau.ac.il)^53^. Individuals were assigned as belonging to a particular cluster with an assignment probability (Q) of ≥ 0.8, with individuals with an assignment probability of 0.2 < Q < 0.8 being classified as ‘admixed’^54,55^). We used two other alternative methods to Bayesian clustering algorithms in order to identify the number of clusters and to characterize the population structure. The first one is discriminant analysis of principal components (DAPC), a multivariate method to identify clusters and describe genetic structure in biological populations, implemented in ADEGENET package for R^56^. The optimal number of clusters K_DAPC_ is chosen as the minimum number of clusters after which the Bayesian information criterion (BIC) increases or decreases by a negligible amount^56^. Results were visualized as scatter plots in ADEGENET.

The second method is a multilocus maximum likelyhood algorithm^57^ that partitions a collection of genotypes into K groups in an iterative approach, implemented in FLOCK v. 3.1^58^. We applied 20 re-allocations for the algorithm, with 50 runs for each K and a LLOD threshold score of 0. The optimal number of clusters, K_F_, was determined by ad hoc “stopping” rules^58^.

## Results

### Ecological and spatial assessments

Overall, 27.7% of the analysed river network in this study were found suitable for *A. torrentium* and *A. bihariensis*, clustered in 15 groups according to the ecological cost (Fig. 2). The range of values for each variable in the cells of the river network where the species were present can be found in Table 1. The random forest classifier out-of-bag estimate of the error rate was 28.6%, with error rates per class of 39.8% for absences and 14% for presences, respectively. An explanation for the model’s appearance to be under-predicting absences may be that some of the absences in the original data set may not have been caused by inadequate habitat conditions, but by various other factors (i.e. field detectability). A potential consequence of this behaviour could be an estimation of slightly wider suitability ranges, but the very good agreement with the observed occupied ranges suggests that this is unlikely. The ecological costs per cell ranged from 0 to 0.773 in the entire network, the distribution of values is provided in Figure S2.

**Figure 3:**
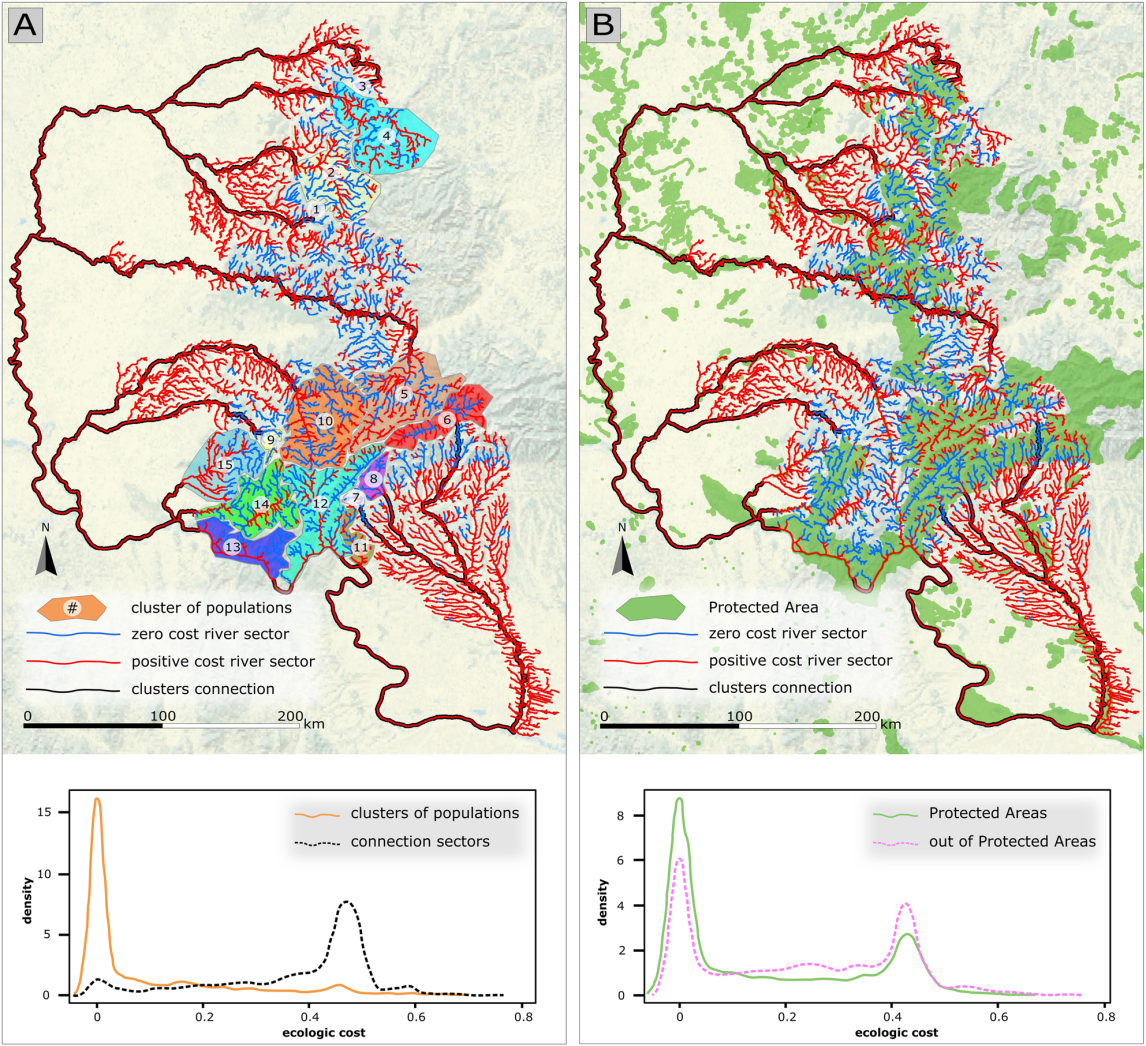
The river network corresponding to crayfish population clusters according to the ecological cost **(A)** and protected areas **(B)**. The distribution of ecological costs on the river network within and between clusters of populations, and inside and outside of protected areas, is presented below maps **(A)** and **(B)**, respectively.

Of the total analysed network, 30.4% were found inside a PA (Fig. 3). Table 2 summarises the basic statistics for the river network in this study. Analysing the ecological cost in the network, we found that for the network situated inside the 15 population clusters, the cost is significantly smaller compared to the cost associated to the network interconnecting them (Mann–Whitney, p < 2.2 × 10^–16^) (Fig. 3A). The ecological cost in the network situated in PAs was significantly smaller compared to that in the network outside PAs (Mann–Whitney, p < 2.2 × 10^–16^). Moreover, a significantly higher suitable network length was found in PAs (39.6%) than outside PAs (22.5%) (Fig. 3B).

**Table 2:** Structure of the river network according to suitability and overlap with PAs within each ecological cluster and on the interconnecting paths; the contribution of each variable to the overall ecological cost, computed as percentage of the total number of raster cells on the cluster’s interconnecting network for which the variable cost was positive (FFP—flash flood potential; RWQ—remote water quality; ALT—altitude; MMT—multiannual mean temperature).

Cluster	% suitable	% PA	Mean cost	% cost per variable			
				FFP	RWQ	ALT	MMT
1	84.9	61.22	0.061	0	100	0	0
2	53.17	39.24	0.142	0.04	93.77	4.46	6.15
3	87.57	91.52	0.048	0	100	0	0
4	40.8	26.11	0.127	0	74.51	13.9	24.84
5	39.56	55.6	0.141	0.3	58.37	38.23	40.73
6	37.75	19.6	0.108	0.54	38.07	56.42	62.27
7	82.58	100	0.033	0	100	0	0
8	83.1	100	0.026	0	77.90	14.36	22.1
9	95.51	25	0.009	0	60.71	14.29	39.29
10	61.47	38.68	0.072	0	70.38	23.17	29.35
11	36.48	37.15	0.161	0	94.65	63.05	63.58
12	61.85	51.69	0.073	1.31	59.68	55.11	59.48
13	55.78	81.66	0.151	0	80.06	96.04	95.96
14	64.54	48.35	0.068	0	69.85	17.21	28.08
15	36.9	24.34	0.173	0	88.13	62.86	77.45
All	52.27	43.57	0.110	0.23	72.08	37.09	43.51
Connect	3.23	51.12	0.383	0	96.86	84.78	66.21

### Genetic diversity

The dataset of 411 individuals was successfully genotyped, with only 1.02% missing data. No loci showed significant linkage disequilibrium tested over all populations after Bonferroni correction. Only three populations significantly differed from HWE expectations after Bonferroni correction: N1 for loci Aas3040 and Aas5, S9 and N6 for locus Tor11. Diversity values for the five loci ranged from 0.489 to 0.802 (H_O_) for *A. torrentium* and from 0.325 to 0.834 (H_O_) for *A. bihariensis*. The expected heterozygosity H_E_ ranged from 0.5196 to 0.755 for *A. torrentium* and from 0.349 to 0.755 for *A. bihariensis*.

No evidence of large allele dropout and scoring errors due to stuttering was found. Also, MicroChecker found evidence of null alleles in four out of 23 populations in one or two loci (N1 at aas3040 and aas5, S5 at ap1 and tor 11, S11 at ap1 and S13 at aas6). FreeNA on the other hand, suggested the existence of null alleles in 55 out of 115 combinations of loci and populations, with values ranging from 0.0001 to 0.1655 (N2 in locus ap1).

**Figure 4:**
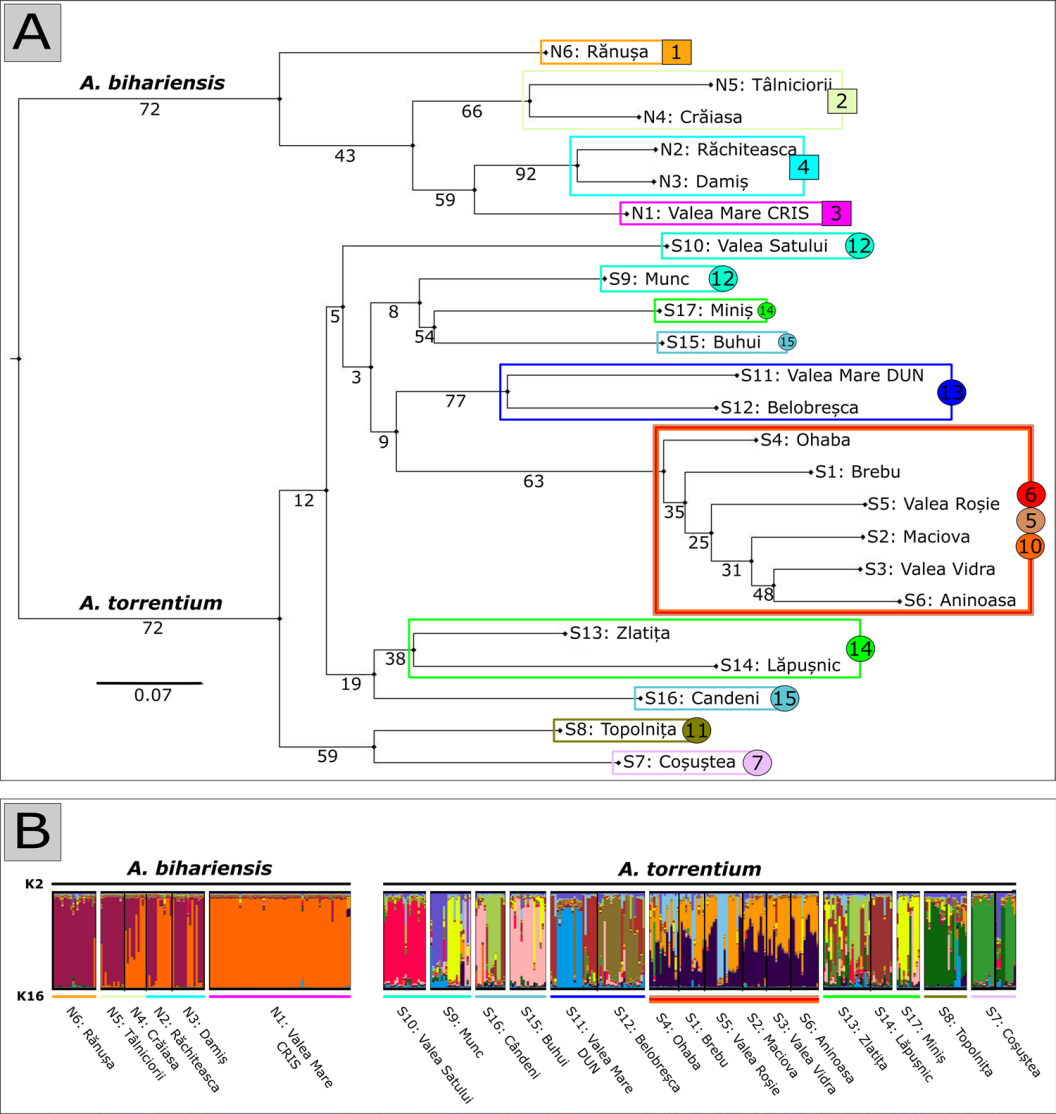
Neighbour-Joining dendrogram constructed with 1,000-bootstrap re-sampling, based on ref.^49^. D_A_ calculated distance showing the relationship among *A. torrentium* and *A. bihariensis* populations from Romanian Carpathians **(A)**; the numbers and coloured boxes in this figure represent the population clusters based on ecological cost. Clustering results obtained using STRUCTURE **(B)**; each individual is represented by a vertical line partitioned into k segments, whose length is proportionate to their membership to each inferred cluster (subdivisions according to species and populations).

The genetic diversity at the population level, measured by the allelic richness (AR), observed heterozygosity (H_O_) and expected heterozygosity (H_E_) varied from 3.91 to 6.98, 0.567 to 0.850 and 0.526 to 0.785, respectively, in *A. torrentium* and from 3.25 to 5.35, 0.485 to 0.752 and 0.503 to 0.680, respectively, in *A. bihariensis*. The inbreeding coefficient (F_IS_) varied from − 0.203 to 0.165 for *A. torrentium* and from − 0.113 to 0.074 for *A. bihariensis* populations (Table S1). One or two private alleles were identified in 13 out of 23 populations, with frequencies varying from 0.023 to 0.125. The highest F_ST_ values were registered between the populations from NW (i.e. *A. bihariensis*) versus populations from SW (i.e. *A. torrentium*). F_ST_ values and F_ST_ ENA values, uncorrected and corrected for null alleles, respectively calculated in FreeNA did not show any significant difference (t = − 0.6257, p = 0.53177). The F_ST_ index for *A. torrentium* populations (Table S2) indicated great population differentiation, with an overall value of 0.191 and pairwise F_ST_ values varying between 0.027 and 0.209. Pairwise F_ST_ values for *A. bihariensis* populations varied between 0.058 and 0.209, and overall F_ST_ had a value of 0.209, indicating high differentiation between analysed populations (Table S2). The Neighbour-Joining tree generated from the Da genetic distances visually illustrates the level of differentiation between *A. torrentium* and *A. bihariensis* as well as further differentiation between the populations of both species (Fig. 4A).

Bayesian analysis in STRUCTURE (Fig. S3) showed that two genetic groups of the investigated crayfish were present in the analysed region (ΔK = 2) when the Evanno method was employed. However, using the Bayesian clustering procedure, the highest likelihoods were obtained for Prob(K) = 16 (Fig. S3). In the first case, the two groups were represented by the two species (Fig. 4). In the second case, with the highest posterior probability Prob(K) = 16, populations of *A. bihariensis* were assigned to two clusters, while the populations of *A. torrentium* were assigned to other 14 different clusters. Overall, 211 individuals out of 411 were assigned to one of the clusters at Q ≥ 0.8, and the remaining 200 proved to be admixed (0.2 < Q < 0.8). The first cluster (for abbreviations, we refer readers to Table S1) was comprised of individuals from N1 (Q > 0.9), and the second cluster was comprised mainly of individuals from N6 and N5 (Q > 0.8). The remaining *A. bihariensis* populations had individuals belonging to both clusters (N3, N2) or were admixed (N4). In the *A. torrentium* populations, only S14, S10 and S15 populations had a higher Q (Q = 0.793, 0.768 and 0.713, respectively), each representing a different cluster. Populations S6, S1, S2, S3 and S4 had all the individuals admixed, and there was a high percentage of admixed individuals in S13 (21 out of 22). The rest of the remaining populations had a proportion of admixed individuals between 28.5 and 77.8%. The two species showed little evidence of mixing.

Evanno method of determining ΔK is best identify the highest level of genetic structure and may not be particularly reliable under complex evolutionary scenarios^59^. And although it is an elegant method from the statistic point of view, it always produces a “solution” for the given data and the solution may not be the best one^60^. The alterative assessment of clusters performed by DAPC revealed K_DAPC_ = 14 inferred clusters, after 40 principal components and 4 discriminant functions were retained (Fig. S4A). The DAPC assignments of individuals from our populations to groups is illustrated in figure S4B. The DAPC scatter plot also clearly delineates the two *Austropotamobius* species populations (see Fig. S4C). The number of clusters inferred in DAPC is smaller than the one found in STRUCTURE (K_DAPC_ = 14 vs Prob(K) = 16). *A. bihariensis* populations are assigned in DAPC to 5 distinct groups instead of 2 as in STRUCTURE Prob K results, with populations N1, N2, N5, N6 genetically differentiated and N3 and N4 with admixed individuals. In the *A. torrentium* populations the number of clusters identified in DAPC is smaller (K_DAPC_ = 9) than in the analysis performed in STRUCTURE (Prob(K) = 14), but the results partially overlap the results performed by the Bayesian clustering aproach. We can see a separation between cluster 1 (k1—including the majority of the samples of S1, S2 and S3), cluster 6 (k6—S5, and S6) and the rest of the clusters with the rest of the *A. torrentium* individuals.

When we performed the multilocus maximum likelyhood proceedure in FLOCK on all populations we reached a “stopping” condition at K_F_ = 2 (with a very long plateau of 50), recognising thus the two species. When the two metapopulations were analysed separately, we obtained an optimum K_F_ = 4 for the ones belonging to *A. bihariensis* and an optimum K_F_ = 2 for the *A. torrentium* populations (see Fig. S5).

Hierarchical AMOVA identified significant geographical structuring and showed an elevated percentage of variation among populations (25%, p = 0.001). Genetic structuring was supported when the STRUCTURE results, 2 and 13 groups, respectively (the number of clusters is lower due to admixed populations) were considered. We also observed a strong genetic structure when we took the genetic variation according to the drainage basins into account (Table S3). Mantel tests of the relationships between genetic and geographic distances proved a moderate correlation and revealed an isolation-by-distance pattern (r = 0.525; p < 0.001).

## Discussion

### The current status of populations

Generally, the results of this study revealed a moderate to high genetic diversity in comparison to other studies on populations of *A. torrentium* from Austria, Switzerland and Germany (e.g. ref.^61^). We observed a strong genetic structuring with no hybridisation between two groups of *Austropotamobius* species populations, suggesting separation that occurred a very long time ago. This result is in agreement with the recent documentation that these populations no longer belong to a single species^18^. The separation was, most likely, driven by a tectonic displacement of the Tisza-Dacia microplate (including the current Apuseni Mountains in NW of Romania) during the early Miocene (ca. 15 Ma) (see ref.^20^).

The clade of *A. bihariensis* revealed high genetic diversity and genetic structuring with up to 4 populations recognised as distinct clusters. The molecular data is supporting the argument for the endemic nature of this species. A homogenous population of *A. bihariensis* was found in the N1 river, presenting lower heterozygosity than other populations. The spatial analysis of this watercourse showed a large ecological distance from the rest of the *A. bihariensis* populations (see Fig. 2). However, genetically, the N1 crayfish were highly similar to the populations in the neighbouring basin (see Fig. 4, populations N1 versus N2, N3). Considering the topography of the area inhabited by populations N1 and N2, namely the fact that the headwaters of both rivers are situated in the same plateau a few hundred meters apart, with human dwellings nearby, we suspect that the N1 population is a result of human (or otherwise mediated) translocations from N2.

The strong genetic differentiation between *A. torrentium* populations indicates both ecological fragmentation and very limited geneflow. The results from the different k-clustering methods suggest a complex structure related to a complicated population history in relation to geographical river basins. Considering that those populations belong to a more recent haplogroup (CSE, sensu ref.^22^) which presumably colonised the Danube basin before and after the Pleistocene glaciations^20,22^, the genetic pattern observed in this study supports the assumption of multiple colonisation episodes in the past, which explains the legacy of high diversity and divergence between neighbouring basins and populations. Spatial analysis of the river network shows a mixture of sectors with zero ecological cost, mainly found in the upper sections of rivers, and sectors with positive ecological cost found especially on the main courses. The RWQ (i.e. water quality) was identified as the most important contributor to the ecological cost, which supports the assumption that the fragmentation pattern is a recent one, most likely resulting from anthropic development^62^.

An unexpected situation was found in a group of populations with a low level of genetic differentiation but considerable ecological separation (see Fig. 4). This is the case for six populations of *A. torrentium* belonging to three different river basins (see Fig. 2): S1, S2, S3 (Timiș River basin), S4 (Strei River basin) and S5 and S6 (northern Jiu River basin). At the same time, a branch of the Jiu River, situated further south, hosts only populations (i.e. S7 and S8) expressing high genetic differentiation from those in the previously mentioned cluster (see Fig. 4, S4). A possible scenario explaining this situation might be that the Carpathians are acting as a natural barrier that historically impeded the colonisation route between the southern and northern populations of the Jiu River. In this respect, we found that there are several cells where the FFP greatly exceeds the species optimum in the Jiu River sector traversing the Carpathians. The molecular cluster of the six populations from the three different basins might be explained by the regressive erosion, documented between the Strei River and the neighbouring tributaries of the Timiș and the Jiu rivers^63^. This phenomenon occurred most likely between Pleistocene glaciation episodes and created temporary connections between tributaries of those river basins^64^, thus leading to the unexpected distribution. An alternative hypothesis, such as the crayfish traveling by land between rivers, is unlikely, as this behaviour was never documented for any *Austropotamobius* species. Expansion driven by human-mediated transport cannot be excluded, although the genetic structure of these populations implies this must have occurred repeatedly in the past for all the three river basins.

### Remarks on conservation

Conservation strategies targeting crayfish are priorities worldwide^17,65^, inside and outside of PAs. The results of this study show the current situation for the two *Austropotamobius* species as being fragmented both ecologically and with respect to gene flow. A deeper analysis of the variables causing ecological fragmentation suggests water quality as a main reason (see Table 2), which is in agreement with other studies^21,66,67^. Water quality deterioration was caused by recent anthropogenic regional development^62^, which is why its effects are not clearly expressed in the observed genetic patterns (see Fig. 4). The nowadays unsuitability of large rivers could be an important issue for the current conservation initiatives, since these rivers were migration pathways in the past. The species distribution in the region is documented as being a result of intense colonisation events after the Paratethys retreat^20^, corresponding to the development of the paleo‐Danube system within the Pannonian Basin^68^.

Fragmentation causes inbreeding and thus homozygosity^69^, which can further increase the chances of offspring being affected by recessive or deleterious traits, leading to a decreased biological fitness of populations and the decline of long-term survival^70^. The artificial gene flow within populations by translocating individuals could be a solution for the improvement of genetic diversity. Still criticised, this method must be carefully applied using the most appropriate gene pool for repopulation to minimise the impact on the original haplotypes’ distribution^71^ and to avoid spread of diseases^72,73^. With respect to habitat adequacy, PAs were found to provide reasonably good conditions for both species. However, the degree of suitability to the species’ requirements drops dramatically on the network connecting the population clusters. Thus, a substantial impediment is found against the communication between these crayfish populations. Even though PAs offer good habitat conditions locally, the long-term protection strategy fails to provide interconnectivity, ensuring the communication corridors between population clusters being a challenging task^74,75^.

Another factor that can influence species distribution in an area is the presence of invasive species and the spread of their associated diseases^76^. As around 20% of the faunal extinctions could be tracked down to invasive species^77^, identification of the main pathways of colonisation for freshwater alien species in Europe is of utmost importance^78^. Our method could help in species risk assessment and identification of probable river pathways (as the ones with the lowest ecological cost) of the highly successful invasive species^78^. In this context, there is a good side of the fragmented pattern found in *A. torrentium* and *A. bihariensis* populations. In the worst scenario of upcoming invasions (see e.g. refs.^31,79^), the fragmented habitats could offer “ark sites” (sensu ref.^80^) of isolated populations, turning into an advantage against unwanted colonisers (see e.g. refs.^16,81^).

## Conclusion and recommendations

Quantifying habitat connectivity by considering the dendritic nature of freshwater networks has been a key element in evaluating the current status in conservation of two sensitive and endangered *Austropotamobius* species in the Carpathians. Combining ecological and genetic approaches, we provide a more complete picture. In this study, without ecological analysis, the fragmentation would have been underestimated, and also without genetic analyses, the translocations and atypical colonisation routes would not have been documented and revealed.

In the light of IUCN Red List of Threatened Species relevant criteria ^82^ such as decreasing population trend^83^, the results of this study might be used to reconsider the “data deficient”^83^ status for *A. torrentium*. Moreover, for the still unrated *A. bihariensis*, a newly described species with few populations and a scarce gene pool, a conservation status assessment is urgently needed.

## Supplementary information

Supplementary Information.
