# Supplementary material for: The future of endangered crayfish in light of protected areas and habitat fragmentation
Source: Sci Rep. 2020 Sep 10;10:14870. doi: 10.1038/s41598-020-71915-w (PMC7483723; doi:10.1038/s41598-020-71915-w)
Supplement: Supplementary file 1 — Supplementary Information. [file 41598_2020_71915_MOESM1_ESM.pdf]

SUPPLEMENTARY INFORMATION for

The future of endangered crayfish in the light of protected areas and habitat fragmentation

Lucian PÂRVULESCU[1*], Elena-Iulia IORGU[2], Claudia ZAHARIA[3], Mihaela C. ION[4,5], Alina SATMARI[6], Ana-Maria KRAPAL[2], Oana-Paula POPA[2], Kristian MIOK[7], Iorgu PETRESCU[2] and Luis-Ovidiu POPA[2]

[1] Department of Biology-Chemistry, Faculty of Chemistry, Biology, Geography, West University of Timisoara, 300115 Timisoara, Romania; [2] "Grigore Antipa" National Museum of Natural History, 011341 Bucharest, Romania; [3] Department of Mathematics, Faculty of Mathematics and Computer Science, West University of Timisoara, 300223 Timisoara, Romania; [4] Institute of Biology Bucharest, Romanian Academy, 060031 Bucharest, Romania; [5] Faculty of Biology, University of Bucharest, 050095 Bucharest, Romania; [6] Department of Geography, Faculty of Chemistry, Biology, Geography, West University of Timisoara, 300223 Timisoara, Romania; [7] Department of Informatics, Faculty of Mathematics and Computer Science, West University of Timisoara, 300223 Timisoara, Romania

Figure S1. Variable importance plot for the Random Forest model according to the mean decrease in Gini index (FFP – flash flood potential; RWQ – remote water quality; ALT – altitude; MMT - multiannual mean temperature).

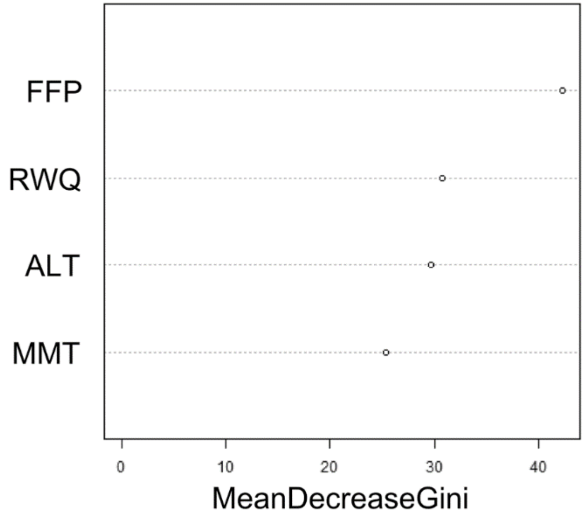

Figure S2. Partial dependence plots for the Random Forest model, illustrating the effect of each variable on crayfish presence probability, after integrating out the other predictors for: (A) altitude – ALT, (B) flash flood potential – FFP, (C) remote water quality – RWQ, (D) multiannual mean temperature – MMT.

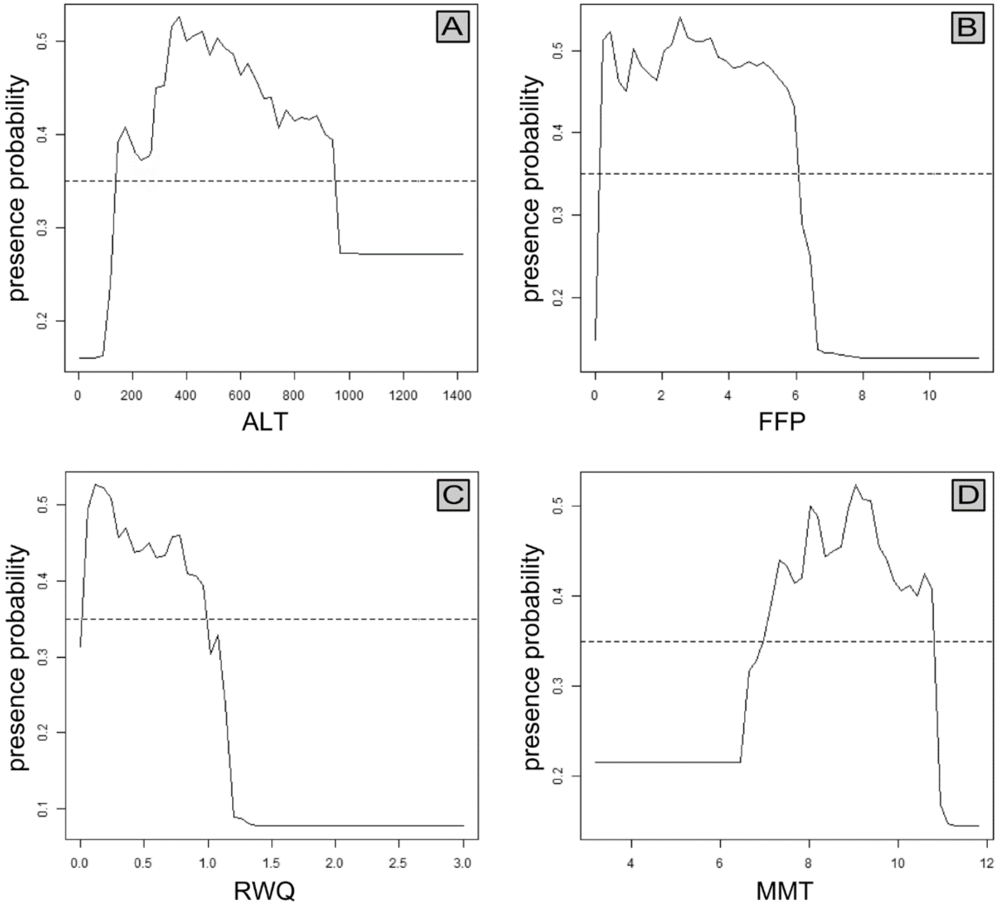

Figure S3. Mean log probability Prob(K) (blue line – right Y axis) and Evanno et al. 2005 ΔK (red line – left Y axis) result from STRUCTURE for *Austropotamobius* species.

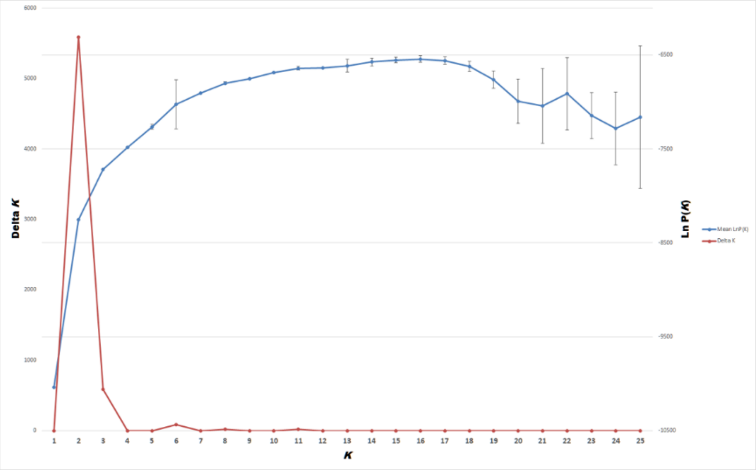

Figure S4. DAPC analysis results for 23 populations of *Austrapotamobius* sp. (A) Inference of the number of clusters in population data. Bayesian information criterion is provided and the chosen number of clusters, K=14, is encircled with red (BICk=14 = 240.3097); (B) Plot of the membership of the *Austropotamobius sp.* populations to the k1-k14 inferred clusters. Rows correspond to populations, while columns correspond to inferred clusters. The size of the squares reflects the number of individuals belonging to one cluster; (C) Scatter plot of DAPC of *Austropotamobius* populations data, showing the first 4 principal components of the DAPC. Clusters are shown by different colors and inertia ellipses, while dots represent individuals. DA and PCA eigenvalues are shown in the inserts at the bottom right and at the top right, respectively, of the scatter plot.

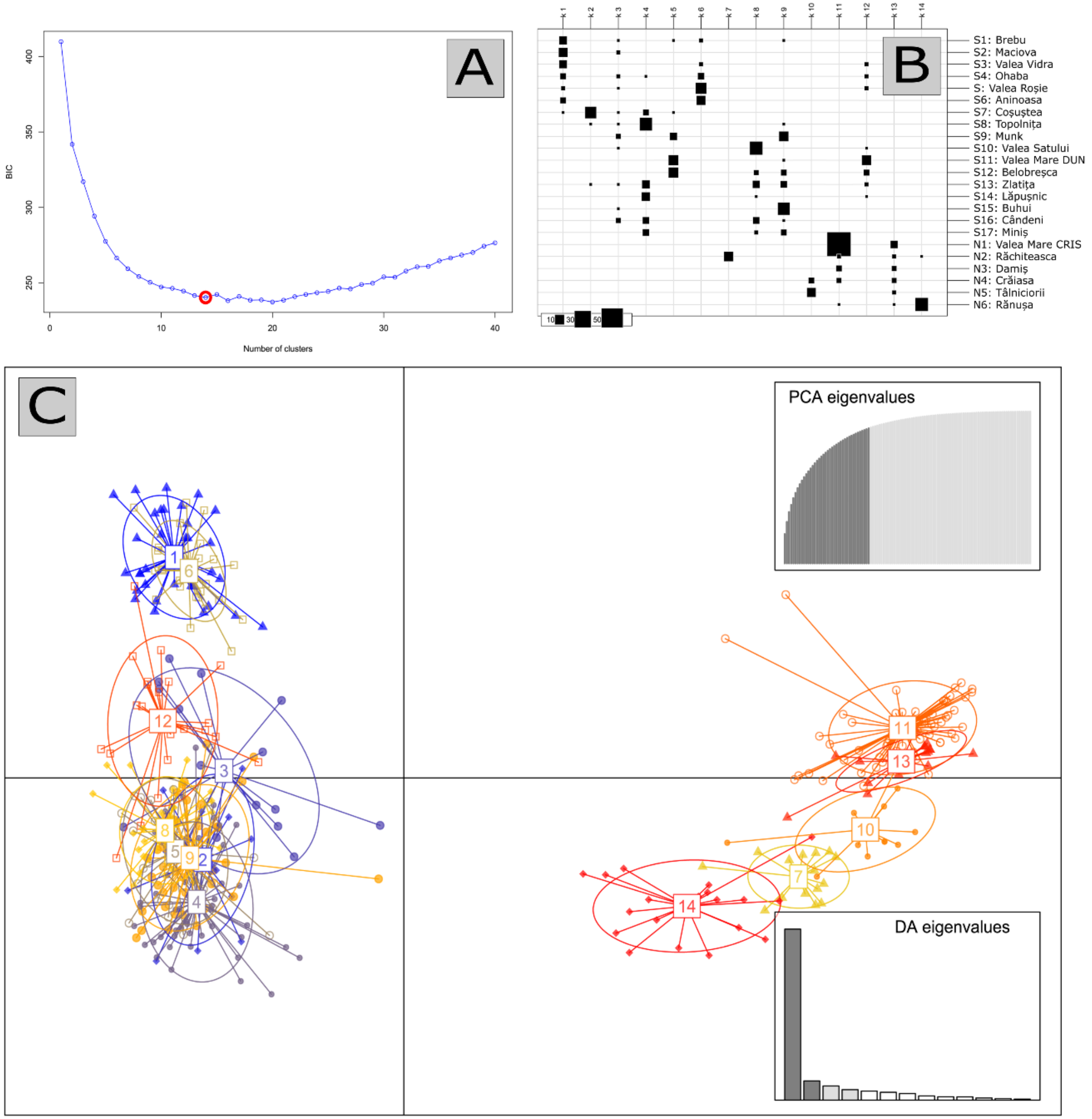

Figure S5. Log likelihood and LLOD values of each population obtained in FLOCK. The optimal number of clusters KF was inferred after the stopping conditions have been met. (A) All the populations KF =2; (B) Only the *A. torrentium* populations KF =2; (C) Only the *A. bihariensis* populations assigned to the inferred clusters KF = 4.

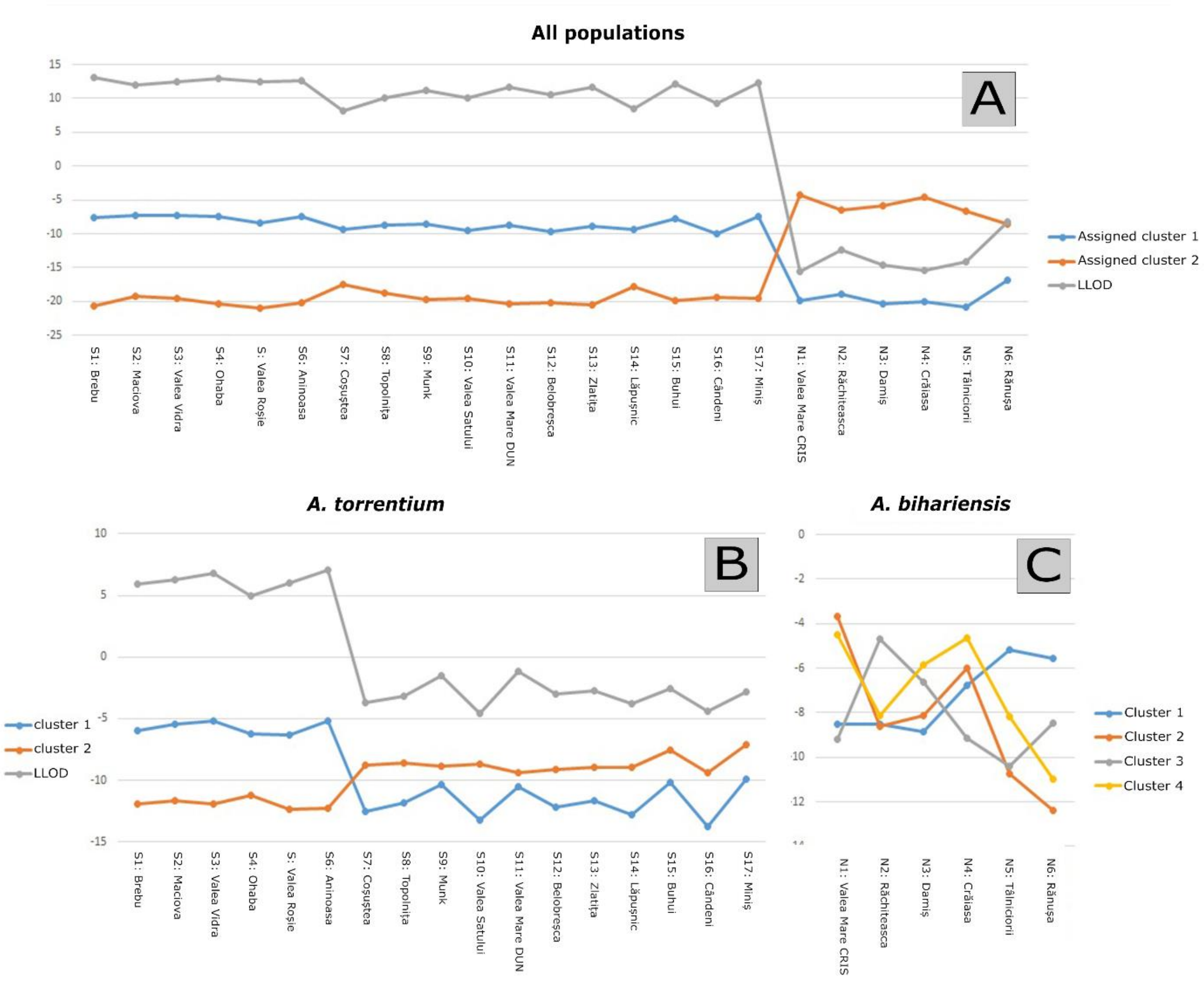

Table S1. Parameters of genetic diversity of investigated crayfish populations (averaged across loci) (N = number of individuals per population, Ne = expected number of alleles, H_O = observed heterozigosity, He = expected heterosigogosity, AR = allelic richness, AP = total number of alleles/number of private alleles, mean F_{IS}).

**Sampling locality**	**Map reference**	**Latitude**	**Longitude**	**N**	**Ne**	**H_O**	**He**	**AR**	**AP**	**F_{IS}**
SW metapopulation (*A. torrentium*)										
**Brebu**	S1	45.234167	22.146389	12	2.954±0.492	0.633±0.122	0.628±0.051	4.64	24/1	0.036
**Maciova**	S2	45.533056	22.198056	11	2.494±0.395	0.691±0.094	0.554±0.074	4.27	22/1	-0.203
**Valea Vidra**	S3	45.418056	22.523611	11	2.449±0.447	0.582±0.110	0.526±0.093	4.10	21/0	-0.06
**Ohaba**	S4	45.507778	22.883056	14	3.040±0.312	0.629±0.069	0.657±0.035	5.12	29/0	0.08
**Valea Roșie**	S5	45.452500	23.370556	18	3.445±0.657	0.567±0.103	0.657±0.076	4.73	26/1	0.165
**Aninoasa**	S6	45.421944	23.290556	13	2.544±0.383	0.677±0.118	0.562±0.079	3.91	21/1	-0.167
**Coșuștea**	S7	44.975278	22.642778	21	4.079±0.718	0.760±0.088	0.715±0.062	6.10	36/1	-0.038
**Topolnița**	S8	44.820556	22.569167	20	3.693±0.860	0.645±0.112	0.655±0.092	6.18	41/2	0.041
**Munc**	S9	45.028598	22.395285	19	4.147±0.626	0.695±0.067	0.740±0.033	6.18	37/1	0.088
**Valea Satului**	S10	44.628890	22.248610	20	4.147±0.944	0.850±0.092	0.714±0.053	6.50	43/2	-0.166
**Valea Mare**	S11	44.741111	21.718056	22	3.769±0.739	0.736±0.109	0.694±0.053	6.34	41/2	-0.038
**Belobreșca**	S12	44.810833	21.497500	22	4.635±0.795	0.781±0.066	0.752±0.048	6.87	44/2	-0.015
**Zlatița**	S13	44.862778	21.485833	22	5.153±0.812	0.697±0.067	0.785±0.034	6.99	42/1	0.135
**Lăpușnic**	S14	44.917500	21.926944	10	3.139±0.463	0.620±0.097	0.644±0.065	5.00	25/0	0.09
**Buhui**	S15	45.064167	21.888889	17	3.034±0.569	0.688±0.093	0.620±0.069	4.64	26/0	-0.078
**Cândeni**	S16	44.946944	21.735556	14	5.606±1.399	0.720±0.120	0.734±0.093	6.89	38/0	0.057
**Miniș**	S17	45.024722	21.823333	11	2.852±0.348	0.782±0.084	0.628±0.045	4.13	21/0	-0.199
NW metapopulation (*A. bihariensis*)										
**Valea Mare**	N1	47.127500	22.610833	66	2.381±0.454	0.485±0.132	0.503±0.102	3.25	23/1	0.044
**Răchiteasca**	N2	47.058333	22.530833	15	3.323±0.697	0.647±0.136	0.633±0.087	4.29	23/0	0.015

**Damiș**	N3	46.866667	22.536667	12	3.078±0.464	0.633±0.120	0.635±0.067	3.85	20/0	0.047
**Crăiasa**	N4	46.546667	22.594722	10	2.405±0.419	0.613±0.085	0.533±0.079	3.38	17/0	-0.097
**Tâlniciorii**	N5	46.416667	22.463333	11	2.236±0.335	0.600±0.106	0.517±0.060	3.84	21/1	-0.113
**Rănușa**	N6	46.439167	22.272778	20	3.705±0.705	0.752±0.088	0.680±0.070	5.35	33/0	-0.08

Table S2. Unbiased estimates of multilocus F_{ST} between all population pairs of the two *Austropotamobius* species based on microsatellites.

**Map reference code**	**S6**	**S12**	**S1**	**S15**	**S16**	**S7**	**S14**	**S2**	**S9**	**S17**	**S4**	**S8**	**S5**	**S3**	**S11**	**S10**	**S13**
	**N1**	**N4**	**N3**	**N2**	**N6**	**N5**											
**S6**	0.000																
**S12**	0.142	0.000															
**S1**	0.066	0.089	0.000														
**S15**	0.178	0.082	0.136	0.000													
**S16**	0.176	0.097	0.142	0.106	0.000												
**S7**	0.175	0.075	0.119	0.158	0.121	0.000											
**S14**	0.209	0.136	0.184	0.164	0.073	0.133	0.000										
**S2**	0.091	0.117	0.046	0.193	0.173	0.126	0.201	0.000									
**S9**	0.124	0.059	0.096	0.045	0.084	0.114	0.113	0.131	0.000								
**S17**	0.160	0.087	0.144	0.075	0.081	0.134	0.100	0.179	0.067	0.000							
**S4**	0.072	0.078	0.030	0.121	0.125	0.102	0.153	0.053	0.081	0.114	0.000						
**S8**	0.196	0.130	0.166	0.138	0.064	0.103	0.065	0.199	0.114	0.082	0.144	0.000					
**S5**	0.027	0.107	0.054	0.144	0.142	0.134	0.174	0.084	0.099	0.124	0.045	0.160	0.000				
**S3**	0.079	0.124	0.052	0.198	0.198	0.131	0.212	0.041	0.152	0.180	0.052	0.208	0.074	0.000			
**S11**	0.118	0.035	0.076	0.101	0.119	0.087	0.151	0.112	0.072	0.104	0.066	0.128	0.088	0.097	0.000		
**S10**	0.165	0.054	0.103	0.113	0.103	0.089	0.154	0.111	0.087	0.106	0.089	0.146	0.120	0.132	0.083	0.000	
**S13**	0.127	0.046	0.097	0.074	0.047	0.064	0.066	0.123	0.054	0.052	0.071	0.054	0.095	0.125	0.057	0.065	0.000
**N1**	0.287	0.238	0.260	0.276	0.226	0.232	0.221	0.268	0.221	0.253	0.241	0.239	0.246	0.304	0.257	0.230	0.210
	0.000																
**N4**	0.281	0.221	0.252	0.264	0.215	0.209	0.214	0.272	0.210	0.250	0.236	0.240	0.243	0.300	0.245	0.219	0.199
	0.058	0.000															
**N3**	0.242	0.176	0.221	0.222	0.180	0.180	0.195	0.243	0.180	0.217	0.207	0.211	0.211	0.256	0.201	0.181	0.166
	0.075	0.061	0.000														
**N2**	0.251	0.177	0.227	0.228	0.183	0.187	0.214	0.252	0.181	0.222	0.214	0.215	0.217	0.269	0.204	0.187	0.170
	0.168	0.165	0.078	0.000													

**N6**	0.235	0.145	0.204	0.199	0.168	0.135	0.188	0.233	0.148	0.197	0.197	0.191	0.203	0.250	0.176	0.171	0.144
	0.209	0.183	0.138	0.088	0.000												
**N5**	0.296	0.223	0.265	0.279	0.224	0.206	0.247	0.303	0.218	0.272	0.261	0.266	0.262	0.315	0.247	0.235	0.210
		0.177	0.092	0.133	0.191	0.143	0.000										

Table S3. Analysis of molecular variance (AMOVA) of spatial genetic diversity in stone crayfish based on microsatellite data.

**Structure tested**	**Variance**	**% variation**	**Fstatistics**	**P**
**One region**				
Among populations	0.564	25%		
Within populations	1.655	75%	FST=0.254	0.001
**Two regions (NW and SW)**				
Among regions	0.433	18%	FRT = 0.177	0.001
Among populations	0.361	15%	FSR = 0.179	0.001
Within populations	1.655	68%	FST = 0.324	0.001
**Sixteen regions (groups according to STRUCTURE analysis)**				
Among regions	0.406	18%	FRT = 0.181	0.001
Among populations	0.184	8%	FSR = 0.100	0.001
Within populations	1.655	74%	FST = 0.263	0.001
**SW hydrographic basins (14 groups): Jiu, Timiș, Ohaba, Belobreșca, Caraș, Nera, Coșuștea, Cerna, Topolnița, Valea Mare DUN, Valea Satului rivers**				
Among regions	0.369	17%	FRT = 0.165	0.001
Among populations	0.211	9%	FSR = 0.113	0.001
Within populations	1.655	74%	FST = 0.260	0.001
